# Advances in Photoplethysmography for Personalized Cardiovascular Monitoring

**DOI:** 10.3390/bios12100863

**Published:** 2022-10-12

**Authors:** Seamin Kim, Xiao Xiao, Jun Chen

**Affiliations:** 1 Department of Physics and Astronomy, University of California, Irvine, CA 92697, USA; 2 Department of Bioengineering, University of California, Los Angeles, CA 90095, USA

**Keywords:** photoplethysmography, cardiovascular disease, biosignal monitoring

## Abstract

Photoplethysmography (PPG) is garnering substantial interest due to low cost, noninvasiveness, and its potential for diagnosing cardiovascular diseases, such as cardiomyopathy, heart failure, and arrhythmia. The signals obtained through PPG can yield information based on simple analyses, such as heart rate. In contrast, when accompanied by the complex analysis of sophisticated signals, valuable information, such as blood pressure, sympathetic nervous system activity, and heart rate variability, can be obtained. For a complex analysis, a better understanding of the sources of noise, which create limitations in the application of PPG, is needed to get reliable information to assess cardiovascular health. Therefore, this Special Issue handles literature about noises and how they affect the waveform of the PPG caused by individual variations (e.g., skin tone, obesity, age, and gender), physiology (e.g., respiration, venous pulsation, body site of measurement, and body temperature), and external factors (e.g., motion artifact, ambient light, and applied pressure to the skin). It also covers the issues that still need to be considered in each situation.

The continuous/intermittent monitoring system based on biosignals can deliver preventive treatment because it can measure abnormal body signals and obtain information such as disease therapy effects [1,2,3,4,5,6,7,8]. Photoplethysmography (PPG), which gets the biosignals related to blood flow with light-emitting diodes and photodetector, is one of the promising monitoring systems, since it enables the diagnosis of diseases directly related to human lives, such as information related to cardiovascular disease (CVD) [9,10,11,12,13]. CVD is a class of chronic conditions and the number one cause of death globally, contributing to more than 17 million deaths [14,15,16]. PPG is one of the noninvasive methods for diagnosing and monitoring these cardiovascular diseases. As it is a noninvasive method, an accurate analysis of the signal obtained from the photodetector is required.

The photodetector collects the transmitted or reflected light depending on the contraction and relaxation of the artery, and we can get the changes in blood flow by analyzing the electrical signals [17]. The periodically changing signals obtain pulsatile information, and changes in light transmittance show changes in erythrocyte orientation [18]. The PPG waveform, a periodic AC pulsatile component of electrical signals by removing the quasi-DC baseline component, has tremendous information to assess cardiovascular health. We can get (i) heart rate, stroke volume, and hypertension by PPG waveform, (ii) blood velocity by the first derivative, and (iii) vascular health and risk for cardiovascular disease by the second derivative [19,20,21,22,23,24,25]. Despite the usefulness of these PPGs, the devices approved by U.S. Food and Drug Administration are limited because of the numerous sources of noise that can impede the output of the PPG [26]. For true health monitoring, these sources of noise and how they affect the waveform of the PPG and its derivatives should be resolved.

The PPG exhibits varied responses in the following conditions: (i) individual variations, (ii) physiological processes, and (iii) external environments. First, we will discuss the factors in the individual variations (e.g., skin tone, obesity, age, and gender) that affect the PPG characteristics. Although the PPG-emitted green light (wavelength: ~550 nm) is relatively insensitive to the melamine content, the PPG signals exhibit a low signal-to-noise ratio in a darker skin tone [27,28]. Obesity is also a primary factor for the PPG system because it can induce multiple changes in the subject’s metabolism (e.g., skin thickness, blood flow, capillary density, etc.) [29,30]. Furthermore, aging that influences the cardiovascular system, skin thickness, and degree of hydration may dampen the PPG resolution [31]. The waveform of the PPG signals can differ in response to the gender-associated physiological differences that involve blood pressure, heart mass, and average heart rate [32]. Therefore, to achieve a high signal-to-noise ratio (SNR), a more optimized PPG system should be established through data library and machine learning [33,34,35,36,37,38].

Next, physiological processes (e.g., respiration, venous pulsations, local body temperature, etc.) mainly affect the PPG waveform. For example, variations in respiratory characteristics produce significant changes in the PPG signals with different intensities, amplitude, and frequencies [39,40,41]. Likewise, venous pulsation yields periodical AC signals resulting from the blood volume changes, which is detrimental to accurate PPG measurement [42,43]. Local body temperature is also found to be positively proportional to the amplitude of the PPG signals [44,45]. Therefore, for precise analysis of the PPG signals, the baseline signals from the physiological processes need to be observed for the post-signal processing.

At last, external factors may modify the noise level of the PPG signals. For example, biomechanical motions with varied frequency ranges induce the fluctuation of the PPG signals [46]. Besides, previous studies state that ambient light limits the accuracy of the PPG measurements with the increased noise level [47]. Thus, these potential external factors need to be controlled and consistent to achieve a more reliable PPG measurement.

In this editorial we have discussed significant challenges to securing the accuracy of the PPG measurements according to the accepted topic. We look forward to advancing the PPG measurements resulting in a precise, personalized technique for the biosignal monitoring system. Moreover, benefiting from its low-power consumption feature, we suggest that energy harvesting technologies and flexible energy storage can be a promising solution for the energy source of PPG-integrated medical devices [48,49,50,51,52,53,54]. Thus, we expect the wearable PPG-based diagnostic system to provide an exceptional clinical experience to patients.
